# To explore the effects of different ways of high intensity interval training on self-control and physical health of college students

**DOI:** 10.3389/fpubh.2025.1550598

**Published:** 2025-05-02

**Authors:** Yan Sun, Yu Wang, Haohan Yu, Kaiwei Cheng, Haoyi Wang, Jingmin Liu

**Affiliations:** ^1^Department of Physical Education, Beijing University of Posts and Telecommunications, Beijing, China; ^2^Department of Physical Education, University of Science and Technology Beijing, Beijing, China; ^3^Division of Sport Science and Physical Education, Tsinghua University, Beijing, China; ^4^Department of Physical Education, Beijing Institute of Technology, Beijing, China

**Keywords:** high-intensity interval training, college students, self-control, physical fitness level, online-offline hybrid teaching model

## Abstract

**Objective:**

The present study aims to investigate the effects of different modes of high-intensity interval training on the self-control and physical fitness levels of college students.

**Methods:**

1. The participants of this study comprised 58 college students, who were randomly divided into three groups: an online experimental group (A = 20), an online-offline experimental group (B = 18), and a control group (C = 20). The Chinese Self-Control Scale (CSCS) and the Physical Fitness Level Test (PHLT) were administered to all participants before and after the intervention, and the test results were then assigned to the respective scores. Group A performed online only, Group B performed online-offline Tai Chi practice, 40 min/1 time/week, 30 min/1 time/week jogging and 40 min/2 times/week high-intensity interval training for 8 weeks, and Group C did not undergo high-intensity interval training. 2. Statistical analysis was performed using SPSS 25.0, including one-way ANOVA for pre-test group differences, paired t-tests for within-group differences, and ANOVA and LSD tests for post-test group differences.

**Results:**

1. A significant improvement in the total score of self-control was observed in group B (*p* < 0.001), while no significant change was observed in groups A and C. 2. A significant enhancement in standing long jump scores was observed in both groups A and B (*p* < 0.01 and *p* < 0.001, respectively), while group A demonstrated a significant enhancement in seated forward bending scores (*p* < 0.05). Additionally, group B exhibited a substantial improvement in 50-m scores (*p* < 0.01). The results of the differences between groups showed significant differences in 50-m scores (*p* < 0.01, B > A; A > C; B > C) and standing long jump scores (*p* < 0.05, B > A; B > C).

**Conclusion:**

1. A variety of high-intensity interval training (HIIT) formats have been demonstrated to enhance the physical fitness and health of university students. 2. The online-offline HIIT methodology has been shown to assist in enhancing the self-control of university students. 3. The online-offline HIIT methodology has the capacity to more effectively improve the self-control ability and physical fitness of college students.

**Suggestion:**

Physical education teachers implement high-intensity interval training programs using online and offline methods to enhance self-control and fitness in university students.

## 1 Introduction

In 1988, the Director-General of the World Health Organization, Dr. Mahler, asserted that “health is not everything, but when you lose it, you lose everything…” This statement elucidates the dialectical relationship among life, career and health. In contemporary society, there has been a surge of interest in the self-control and physical fitness of university students (1–5). This is due to the fact that self-control refers to the ability to inhibit immediate impulses and regulate one’s own behavioral, emotional and cognitive processes (6), which plays an important role in an individual’s academic achievement, social adaptation and psychological health. The level of physical fitness, on the other hand, is directly related to an individual’s quality of life and long-term health.

The dual-systems theory of self-control posits that self-control performance is the result of the interaction between the impulsive system and the self-control system. The information processing process of the impulsive system is automatic, unconscious, and does not rely on psychological resources. Impulsive behavioral tendencies tend to be contrary to health goals in a variety of situations (7), such as various addictive problems (8), suicidal tendencies (9), and states of depression, anxiety, and stress (10). The self-control system is the antithesis of this; therefore, individuals must exercise restraint over impulsive tendencies and enhance self-control. Research has demonstrated that exercise training can enhance self-control across various domains, including resisting temptation, delaying gratification, and adhering to goals (11). It is imperative for college students to effectively regulate their behaviors to achieve satisfactory outcomes across various domains when confronted with diverse demands, such as academic pressures, social activities, and personal interests. This underscores the significance of self-control in promoting the healthy development of college students’ physical and mental well-being. Research has indicated that college students who engage in regular high-intensity interval training demonstrate heightened levels of self-control (12). Nevertheless, the precise mechanisms underlying this relationship remain to be fully elucidated. Potential explanations may include the positive physiological and psychological effects of high-intensity interval training, which indirectly enhances self-control. For instance, high-intensity interval training has been demonstrated to enhance cardiorespiratory fitness, promote fat metabolism (13), improve muscle function (14), and enhance quality of life in university students (15). HIIT also has a positive impact on cognitive functioning in university students, which can improve performance in terms of cognitive flexibility, selective attention, and working memory (16). Furthermore, the enhancement of self-control may be associated with specific psychological factors during high-intensity interval training. A number of studies have demonstrated that HIIT can increase the level of brain-derived neurotrophic factor (BDNF), with plasma BDNF levels showing significant increases after a single session of HIIT. This may assist in enhancing the cognitive function and emotional state of college students. Concurrently, cortisol levels are elevated in conjunction with BDNF, working in synergy to enhance the ability of working memory (17). The enhancement of college students’ self-control can be attributed to HIIT through multiple physiological, psychological and cognitive mechanisms. However, further research is necessary to elucidate the specific mechanisms through which HIIT enhances college students’ self-control.

High-intensity interval training (HIIT) constitutes a training modality whereby exercise is performed at a load of maximal lactate homeostasis or at a load intensity that is greater than or equal to the anaerobic threshold. These periods of exercise are interspersed with low-intensity recovery or rest periods (18). This training modality has garnered significant attention for its time efficiency and substantial enhancement of cardiorespiratory fitness (19–21). The alternating sequence of high-intensity and low-intensity periods has been shown to elicit a substantial caloric expenditure in a relatively brief timeframe, while concomitantly augmenting the body’s metabolic rate (22). A seminal study on high-intensity interval training revealed that the standing long jump, 7-min maximal speed and 20-m round trip run exhibited significant enhancements in the high-intensity interval training group, while the control group demonstrated no substantial changes, thereby suggesting that high-intensity interval training enhances cardiovascular metabolism, speed qualities, and strength and explosive power (23). A notable characteristic of high-intensity interval training is its efficiency in terms of time allocation. The high-intensity interval group required a mere 15% of the training time of the moderate-intensity interval training group to achieve a substantial reduction in cardiovascular risk over a 7-week period. This was accompanied by a significant enhancement in diastolic blood pressure and aerobic capacity (24, 25). Conversely, the appeal and habit-forming potential of high-intensity interval training are more pronounced than those of endurance exercise (26, 27). This finding suggests that, for college students, the utilization of high-intensity interval training methods can be a time-efficient, effective, and efficient approach to physical exercise.

The physical fitness level of an individual is a significant indicator of their health status, involving body composition, physical function, physical quality and other aspects. In the comprehensive evaluation of the physical fitness level of college students, a series of test items and scoring criteria in the National Physical Fitness Standard (2014 edition, hereinafter referred to as the “national test standard”) (28) are usually used for assessment. Quantitative analysis of the results of these test items can lead to an individual’s physical fitness level score, and then to an evaluation of their health status. In a study of college students’ physical fitness testing, it was found that the use of the “national standard” test improved the physical fitness level of senior college students, greatly increased the importance of physical exercise, and could significantly supplement the existing public physical education (29). Consequently, physical education teachers at the collegiate level are encouraged to leverage the benefits of high-intensity interval training (HIIT), namely its time efficiency and capacity to enhance physical fitness. This approach not only addresses the need for timely physical education but also optimizes the utilization of classroom time, thereby facilitating the enhancement of college students’ overall development. It is noteworthy that HIIT has been found to be more engaging and enjoyable than other training modalities (30). This quality, in turn, has been demonstrated to stimulate students’ enthusiasm for learning and their willingness to participate, thus rendering physical education more dynamic and appealing.

## 2 Subjects and methods

### 2.1 Study subjects

A total of 86 students enrolled in the Physical Education and Fundamentals class, taught by the same instructor, were considered for participation in the study. The study was conducted during the autumn semester of 2023 at Beijing University of Posts and Telecommunications (BUPT). The inclusion criteria included the following: no regular exercise training for at least 2 months per year, no smoking or drinking habits, no recent history of infectious diseases, no chronic illnesses such as hypertension, diabetes mellitus, or other clearly diagnosed illnesses, and no history of familial hereditary diseases. Following a thorough evaluation of the candidates, 75 volunteers were deemed to meet the necessary criteria for participation. The team of teachers provided a comprehensive explanation of the experiment’s purpose and methodology to the 75 subjects during the theoretical course section of Physical Education and Fundamentals. Following the detailed explanation of the study’s purpose and methodology, 58 out of the 75 volunteers consented to participate in the eight-week “Physical Fitness Exercise” program, which incorporated high-intensity interval training. The consent form was signed by the students’ representatives.

### 2.2 Research methods

#### 2.2.1 Self-Control Scale

The Chinese version of the Self-Control Scale (SCS) was administered and collected by means of a questionnaire. The scale was originally developed and published by Tangney in the United States in 2004. Subsequent to this, Alexander Unger, Bi Chongzeng, and others translated, validated, and revised the Chinese version of the scale in 2016 (31). The revised scale was validated for use in China. The Chinese version of the scale retained the 36 questions of the English version and was scored on a 5-point scale, with 1 being very non-compliant and 5 being very compliant. The scale is comprised of five dimensions: general ability to self-regulate, thoughtfulness, healthy habits, work ethic, and reliability. The scoring method employed for the scale involved assigning positive values to questions 1, 5, 7, 13, 15, 18, 22, 26, 27, and 36, while negative values were assigned to all other questions. The questionnaire was designed to be concise, eschewing the use of sensitive terms and leading questions. The reverse scoring and the post-collection screening and analysis of the data, which was then subjected to outlier removal and other data refinement, served to mitigate the potential impact of the social desirability effect on the subjects. Consequently, the correlation between the total score and the individual’s self-control was positive.

#### 2.2.2 Assessment of physical fitness levels

The test is conducted in strict accordance with the requirements of the Chinese Ministry of Education’s National Physical Fitness Standard for Students (2014 edition). Body composition: BMI, calculated by dividing weight (kg) by height (m^2^). Physical function: Lung capacity, which tests the functional status of the human respiratory system; and Physical fitness, the test indexes include 50 m, standing long jump, seated forward bending, sit-ups (for females), pull-ups (for males), 800 m (for females), and 1,000 m (for males). The 50-metre test (with an accuracy of 0.1 seconds) assesses the development of students’ speed, agility and the flexibility of their nervous system. The standing long jump (with an accuracy of 0.1 centimetres) primarily measures the development of the explosive power of the muscles in the lower limbs and the coordination of the body when jumping forward. The test of pull-ups and sit-ups responds to the muscle power of the upper limbs and the abdominal area; the test of seated forward bending reflects the flexibility of the joints and the muscles; the test of 800 and 1,000 m (accurate to 0.001 s) tests students’ flexibility. This test, which takes 0.001 s, evaluates the development level of students’ endurance qualities, with a particular focus on the function of the cardiovascular, respiratory system and muscular endurance.

A student physical fitness tester was utilized to assess the physical fitness of the participants before and after the intervention, as required. The pre-test was scheduled to be administered uniformly in the Physical Education Fundamentals class during the second teaching week. Prior to the administration of the physical fitness test, the standardized instrument was meticulously calibrated and debugged by the teaching staff to ensure optimal functionality and the accuracy of the test data. Concurrently, the utilization of a single testing instrument ensured consistent measurement results, thereby enhancing the reliability of the test outcomes. The testers adhered to the instrument’s operating instructions, ensuring its correct usage and providing guidance to subjects during the test. The utilization of a uniform standard instrument for the physical fitness test engendered the comparability of results across diverse groups.

Following the conclusion of the test, the test scores were assigned to correspond to the scores of the National Physical Fitness Standard for Students (2014 edition) of the Ministry of Education of the People’s Republic of China (Table 1).

**Table 1:** Physical health test indicators equipment.

	Measurement indicator	Testing equipment/materials
Body shape	Height and standard weight (index)	Height and weight testing instrument
Body function	Vital capacity	Vital capacity tester
Physical fitness	Pull-ups (male) (count)	Pull-up bar
	1-min sit-ups (female) (count)	Stopwatch
	Standing long jump	Long jump tester
	Sit-and-reach	Sit-and-reach tester
	50-meter sprint	Stopwatch

Control of experimental variables:

1. Selected students were from the 6th–7th and 8th–9th class sessions of the freshmen physical education foundation course.
2. Testing was conducted between 13: 00–16: 10.
3. Location: Changping Campus.
4. Indoor and outdoor temperature: 15–20°C, with a slight breeze of 1–3 levels outdoors.
5. Fatigue control: there was a 2–3 day interval between tests.
6. Test conductor: the same teacher conducted all tests.
7. Procedure: all participants followed the same warm-up, testing, and stretching routine.

#### 2.2.3 Experimental grouping

Fifty-eight subjects were randomized into three groups: an online experimental group (A, *n* = 20), an online-offline experimental group (B, *n* = 18), and a control group (C, *n* = 20). The original hypothesis was that all groups would have equal means. An alternative hypothesis was formulated, proposing that at least one group’s mean would differ from those of the other groups. A one-way ANOVA analysis was employed to compare the groups and ascertain if there was a significant difference between them. The test objectives encompassed age, height, weight, and BMI. The findings revealed that the samples from the various groups exhibited consistency in all of the aforementioned objectives, with no significant differences observed (*p* > 0.05). This finding serves to validate the original hypothesis. This indicates that the mean values of the online experimental group, the online-offline experimental group and the control group in terms of age, height, weight, BMI and BMI score are relatively similar. The specific details can be found in Table 2.

**Table 2:** Analysis of variance between groups A, B, and C.

Measure (units)	(Mean ± SD) within each of the three groups			*F*	*p*
	Group A (*n* = 20)	Group B (*n* = 18)	Group C (*n* = 20)		
Age	18.70 ± 0.73	18.56 ± 0.62	18.65 ± 0.75	0.204	0.816
Height (cm)	170.85 ± 8.57	171.00 ± 8.27	169.65 ± 8.40	0.151	0.86
Weight (kg)	61.40 ± 11.79	60.56 ± 9.83	66.89 ± 15.25	1.457	0.242
BMI	20.94 ± 3.08	20.62 ± 2.22	22.33 ± 2.87	2.098	0.132

*p > 0.05. cm, centimeter; kg, kilogram.*

As demonstrated in Table 3, the chi-square test (cross-tabulation analysis) was employed to ascertain the existence of an association between the two categorical variables “gender” and “grade” across the various groups. The primary hypothesis was as follows: The null hypothesis is that there is no difference between the observed and expected values, i.e., there is no association between the variables. The alternative hypothesis is as follows: Conversely, the alternative hypothesis postulates the presence of a discrepancy between the observed and expected values, thereby signifying an association between the variables. The results of the analyses demonstrate that there is no significant difference (*p* > 0.05) between groups for the categorical variables “gender” and “grade.” This finding serves to validate the original hypothesis.

**Table 3:** Cross-sectional (chi-square) analysis of the variables “gender” and “grade” in groups A, B, and C.

Measure		Group (%)			Total	*p*
		Group A1 (*n* = 20)	Group A2 (*n* = 18)	Group B (*n* = 20)		
Gender	Male	10 (50.00)	9 (50.00)	10 (50.00)	29 (50.00)	1.000
	Female	10 (50.00)	9 (50.00)	10 (50.00)	29 (50.00)	
Total		20	18	20	58	
Grade	1	20 (100.00)	17 (94.44)	17 (85.00)	54 (93.10)	0.394
	2	0 (0.00)	1 (5.56)	2 (10.00)	3 (5.17)	
	3	0 (0.00)	0 (0.00)	1 (5.00)	1 (1.72)	
Total		20	18	20	58	

*p > 0.05. cm, centimeter; kg, kilogram.*

The findings of this investigation demonstrate that there is no correlation between the categorical variables of “gender” and “grade.” It is therefore recommended that, in the formulation of educational policies, teaching methods and curriculum design, greater emphasis be placed on the individual differences and learning needs of students, rather than categorizing them solely on the basis of gender and grade. Rather than relying on such simplistic categorizations, educators are encouraged to adopt a more nuanced approach that acknowledges the diverse learning needs and individual differences among students. For instance, teaching methods can be rendered more adaptable, with individualized instruction based on students’ learning styles and interests, as opposed to uniform teaching arrangements based on gender and grade level.

### 2.3 Statistical analysis

Data are presented as mean ± standard deviation. SPSS 25.0 software was used for:

One-way ANOVA to analyze pre-test group differences.

Paired *t*-tests to assess within-group differences across the three test groups.

ANOVA and LSD methods to examine post-test differences between groups.

## 3 Research process

### 3.1 Pre-test

As demonstrated in Table 4. There was no significant difference (*p* > 0.05) between subjects in groups A, B and C in all scores of total self-control and fitness level before high-intensity interval training.

**Table 4:** The result of one-factor analysis of variance.

Measure (units)	(Mean ± Standard Deviation) within each of the three groups			*F*	*p*
	Group A1 (*n* = 20)	Group A2 (*n* = 18)	Group B (*n* = 20)		
Age	18.70 ± 0.73	18.56 ± 0.62	18.65 ± 0.75	0.204	0.816
Height (cm)	170.85 ± 8.57	171.00 ± 8.27	169.65 ± 8.40	0.151	0.86
Weight (kg)	61.40 ± 11.79	60.56 ± 9.83	66.89 ± 15.25	1.457	0.242
Self-control (score)	109.75 ± 12.37	104.28 ± 9.35	102.00 ± 13.83	2.166	0.124
Vital capacity (score)	80.30 ± 10.44	82.67 ± 12.85	84.25 ± 17.39	0.409	0.666
50 m (score)	68.20 ± 14.97	68.44 ± 7.18	68.15 ± 13.07	0.003	0.997
Standing long jump (score)	64.60 ± 18.51	64.00 ± 15.22	65.20 ± 23.31	0.018	0.982
Sit-and-reach (score)	76.90 ± 9.77	83.50 ± 11.60	76.65 ± 13.82	1.999	0.145
800 m/1,000 m (score)	71.90 ± 14.56	72.44 ± 13.26	74.60 ± 13.61	0.211	0.81
Sit-ups/pull-ups (score)	39.25 ± 41.13	51.72 ± 40.27	39.90 ± 37.25	0.587	0.56
Total score of physical fitness test	72.40 ± 8.38	75.17 ± 7.47	73.15 ± 8.93	0.556	0.577

*P > 0.05. cm, centimeter; kg, kilogram.*

As demonstrated in Table 5, non-parametric tests were utilized to examine the variability of ordinal numbers on sit-up/pull-up scores for a single item, as illustrated in the above table. Ordinal numbers encompass more than two groups, and consequently, they were analyzed employing the Kruskal-Wallis test statistic. The ordinal number samples were found not to be statistically significant (*p* > 0.05) for all sit-up/pull-up scores, suggesting that the ordinal number samples are consistent for all sit-up/pull-up scores and that variability is not present.

**Table 5:** Results of non-parametric test analysis.

Measure (score)	The median between groups M (P25, P75)			The H value of the Kruskal-Wallis test statistic	*p*
	Group A1 (*n* = 20)	Group A2 (*n* = 18)	Group B (*n* = 20)		
Sit-ups/pull-ups	33.000 (0.0, 74.0)	57.000 (0.0, 91.3)	56.000 (0.0, 73.0)	1.629	0.443

*p > 0.05.*

To conclude the analysis, the analysis using the Kruskal-Wallis test statistic shows that none of the samples with different serial numbers will show a significant difference for sit-up/pull-up scores.

### 3.2 High-intensity interval training program

Each high-intensity interval training session for Groups A and B comprised a 5-min acclimatization session, a 30-min main exercise component, and a 5-min relaxation activity. The training sessions were administered twice a week on Tuesdays and Saturdays. Group A engaged in online training facilitated by the instructor, who provided the classroom group with their contact information for monitoring attendance. Group A then convened for group training sessions. Group B engaged in offline training in a group, with sessions held in the classroom. The study is conducted 16 times over an 8-week period. Group C does not perform high-intensity interval training.

Group A (*n* = 20): 40 min of Yang’s taijiquan offline and 40 min of high-intensity interval training online on Tuesdays; 30 min of jogging offline (at a pace of 10 min/km or less) and 40 min of high-intensity interval training online on Saturdays.

Group B (*n* = 20): 40 min of Yang’s taijiquan and 40 min of high-intensity interval training offline on Tuesdays; 30 min of jogging offline (at a pace of up to 10 min/km) and 40 min of high-intensity interval training online on Saturdays.

Groups A and B performed long-interval training, which included 75 s of speed and agility class, as well as four unarmed movements. These were the cross quadrant jump, step open and close, *in situ* high leg raise, and square contact run. It is important to note that each movement corresponded to 100% of the maximum oxygen uptake, resulting in a heart rate that exceeded 150 beats per minute. A 180 s of incomplete recovery, each recovery using operatic stretching, dynamic stretching, the maximum oxygen uptake of 50–60%, heart rate reaches 110–130 beats per minute; 180 s of incomplete recovery, each recovery using operatic stretching, dynamic stretching, the maximum oxygen uptake of 50–60%, heart rate up to 110–130 beats per minute. The subsequent 50-s interval training focused on hip and leg resistance and comprised four distinct movements: *in situ* deep squat, semi-squat jump up, alternating squatting on both the left and right sides, and alternating lunge squatting with support leg lifted. The intensity of each exercise corresponded to a maximal oxygen uptake of 70–80%, with a heart rate ranging from 130 to 150 beats per minute. Following this, a 50-s period of incomplete recovery was initiated, utilizing in situ stepping and parallel stepping, both of which were performed at a maximal oxygen uptake of 50–60% and a heart rate between 110 and 130 beats per minute.

Group C (*n* = 18): The exercise routine comprised 40 min of Yang’s taijiquan offline and 30 min of jogging offline on Tuesdays (at a pace of 10 min per kilometer or less). No high-intensity interval training of any kind was performed. VOmax was monitored during exercise by subjective measurements and combined with objective measurements of parameters such as heart rate to ensure that participants achieved the target exercise intensity. Music with varying intensities was used to continuously encourage participants to keep pace and maintain their speed. Consequently, Groups A and B were requested to undertake the Borg 6–20 RPE Scale (32) during intervals, in addition to wearing exercise bracelets while engaging in high-intensity interval training (the system under scrutiny is an exercise load monitoring system of the SunFitLlik brand, model 550).

## 4 Results of the study

### 4.1 Effects of high-intensity interval training on self-control

A comparative analysis of the total self-control scores of the three groups of college students revealed an improvement in mean values before and after the experiment. Of particular note is the Group B, which demonstrated a significant decrease in mean values from the pre-test to the post-test level (*t* = −4.783, *p* = 0.000). A further analysis revealed that the mean value of the pre-test self-control (104.28) was significantly lower than the mean value of the post-test self-control (114.83). No further statistical disparities were identified between the two groups.

### 4.2 Effects of High-intensity interval training on college students’ physical fitness levels

As demonstrated in Table 6, Group A exhibited a 0.01 level of significance between the pre-test standing jump and the post-test standing jump (*t* = −3.810, *p* = 0.001). The specific comparison difference indicates that the pre-test mean (64.60) will be significantly lower than the post-test mean (72. 85); the pre-test seated forward bending and the post-test seated forward bending showed a 0.05 level of significance between the pre-test and the post-test seated forward bending (*t* = −2.657, *p* = 0.016), as well as the specific comparison difference showing that the pre-test mean (76.90), will be significantly lower than the post-test mean (82.15).

**Table 6:** Results of paired *t*-test analysis—group A.

Measure (score)	(Mean ± SD)		Difference	*t*	*p*
	Before	After			
Self-control (score)	109.75 ± 12.37	109.95 ± 7.63	−0.2	−0.072	0.944
BMI (score)	94.00 ± 11.42	95.00 ± 8.89	−1	−1	0.33
Vital capacity (score)	80.30 ± 10.44	83.10 ± 11.73	−2.8	−0.895	0.382
50 m (score)	68.20 ± 14.97	71.75 ± 8.37	−3.55	−1.15	0.264
Standing long jump (score)	64.60 ± 18.51	72.85 ± 17.66	−8.25	−3.81	0.001**
Sit-and-reach (score)	76.90 ± 9.77	82.15 ± 10.73	−5.25	−2.657	0.016*
800 m/1,000 m (score)	71.90 ± 14.56	70.50 ± 9.94	1.4	0.803	0.432
Sit-ups/pull-ups (score)	39.25 ± 41.13	38.50 ± 40.13	0.75	0.686	0.501
Total score of physical fitness test	72.40 ± 8.38	74.47 ± 7.59	−2.06	−2.904	0.009**

*P < 0.05, **P < 0.01.

As demonstrated in Table 7, Group B exhibited a 0.01 level of significance between the pre-test and post-test 50 m (*t* = −2.965, *p* = 0.009), in addition to specific comparative differences. As illustrated, the pre-test mean (68.44) would be significantly lower than the post-test mean (79. 00); the pre-test and post-test standing jump scores showed a 0.01 level of significance between the pre-test and post-test standing jump scores (*t* = −4.756, *p* = 0.000), as well as the specific comparison differences show that the pre-test mean (64.00), would be significantly lower than the post-test mean (85.33).

**Table 7:** Results of paired *t*-test analysis—group B.

Measure (score)	(Mean ± SD)		Difference	*t*	*p*
	Before	After			
Self-control (score)	104.28 ± 9.35	114.83 ± 4.69	−10.56	−4.783	0.000***
BMI (score)	96.67 ± 10.29	95.56 ± 10.97	1.11	1	0.331
Vital capacity (score)	82.67 ± 12.85	82.94 ± 15.81	−0.28	−0.099	0.923
50 m (score)	68.44 ± 7.18	79.00 ± 14.05	−10.56	−2.965	0.009**
Standing long jump (score)	64.00 ± 15.22	85.33 ± 11.92	−21.33	−4.756	0.000***
Sit-and-reach (score)	83.50 ± 11.60	86.22 ± 11.50	−2.72	−2.058	0.055
800 m/1000 m (score)	72.44 ± 13.26	72.67 ± 4.28	−0.22	−0.088	0.931
Sit-ups/pull-ups (score)	51.72 ± 40.27	51.00 ± 38.06	0.72	0.113	0.911
Total score of physical fitness test	75.17 ± 7.47	77.18 ± 6.98	−2.01	−2.084	0.053

**P < 0.01, ***P < 0.001.

As demonstrated in Table 8, Group C exhibited a statistically significant relationship (*p* = 0.020) between the pre-test seated body forward flexion and the post-test seated body, as indicated by the *t*-value of −2.541. Furthermore, a specific comparison of the differences reveals that the pre-test mean (76.65) would be significantly lower than the post-test mean (81.70).

**Table 8:** Results of paired *t*-test analysis—group C.

Measure (score)	(Mean ± SD)		Difference	*t*	*p*
	Before	After			
Self-control (score)	102.00 ± 13.83	102.25 ± 13.49	−0.25	−0.128	0.9
BMI (score)	91.00 ± 13.73	92.00 ± 11.96	−1	−1	0.33
Vital capacity (score)	84.25 ± 17.39	83.80 ± 14.79	0.45	0.157	0.877
50 m (score)	68.15 ± 13.07	64.35 ± 7.38	3.8	1.283	0.215
Standing long jump (score)	65.20 ± 23.31	68.60 ± 23.08	−3.4	−1.002	0.329
Sit-and-reach (score)	76.65 ± 13.82	81.70 ± 10.98	−5.05	−2.541	0.020*
800 m/1,000 m (score)	74.60 ± 13.61	72.70 ± 10.97	1.9	0.966	0.346
Sit-ups/pull-ups (score)	39.90 ± 37.25	37.90 ± 39.76	2	0.368	0.717
Total score of physical fitness test	73.15 ± 8.93	76.06 ± 7.39	−2.91	−2.803	0.011*

*P < 0.05.

Groups A, B and C exhibited no statistically significant alterations in other physical qualities, as well as body composition and physical functioning, with the exception of the aforementioned items.

The study’s findings revealed a substantial improvement in seated forward bending among group C members. After 8 weeks of high-intensity interval training, group A demonstrated significant improvements in standing jump and seated forward bending, while group B exhibited notable advancements in the 50-m sprint and standing long jump.

### 4.3 Posttest intergroup difference results for groups A, B, and C

The application of analysis of variance (ANOVA) to the study of differences between post-test groups A, B and C reveals that the data demonstrate significance (*p* < 0.05) for a total of three items, namely the total self-control score, 50 m score and vertical jump score. This finding indicates the presence of a differentiation between the groups, which can be further investigated using specific *post-hoc* test analysis (Table 9).

**Table 9:** Results of posttest intergroup differences for groups A, B, and C.

Measure (score)	(Mean ± SD) within each of the three groups			*F*	*p*
	Group A1 (*n* = 20)	Group A2 (*n* = 18)	Group B (*n* = 20)		
Self-control	109.95 ± 7.63	114.83 ± 4.69	102.25 ± 13.49	8.573	0.001***
BMI	95.00 ± 8.89	95.56 ± 10.97	92.00 ± 11.96	0.625	0.539
Vital capacity	83.10 ± 11.73	82.94 ± 15.81	83.80 ± 14.79	0.02	0.98
50 m	71.75 ± 8.37	79.00 ± 14.05	64.35 ± 7.38	9.788	0.000***
Standing long jump	72.85 ± 17.66	85.33 ± 11.92	68.60 ± 23.08	4.217	0.020*
Sit-and-reach	82.15 ± 10.73	86.22 ± 11.50	81.70 ± 10.98	0.946	0.395
800 m/1,000 m	70.50 ± 9.94	72.67 ± 4.28	72.70 ± 10.97	0.384	0.683
Sit-ups/pull-ups	38.50 ± 40.13	51.00 ± 38.06	37.90 ± 39.76	0.657	0.522
Total score of physical fitness test	74.47 ± 7.59	77.18 ± 6.98	76.06 ± 7.39	0.658	0.522

*P < 0.05, ***p < 0.001.

The LSD method was utilized to analyze the disparities between the post-test groups, and the findings indicated that self-control exhibited a 0.01 level of significance (*F* = 8.573, *p* = 0.001). The comparison of the group mean scores yielded the following results: “experimental group A > control group C; experimental group B > control group C,” thereby suggesting that online and offline high-intensity interval training is reported to enhance college students’ self-control. In terms of physical fitness, the score of 50 m demonstrated a 0.01 level of significance (*F* = 9.788, *p* = 0.000), and the comparison of group mean scores was “B experimental group>A experimental group; A experimental group>C control group; B experimental group>C control group.” The score of the standing jump demonstrated a 0.05 level of significance (*F* = 4.217, *p* = 0). The results demonstrated that the experimental group (B) exhibited superior performance compared to the control group (C). This observation was further supported by statistical analysis, which revealed a significant difference in mean scores between the experimental groups (*p* = 0.000). The experimental group demonstrated a higher level of effectiveness in enhancing various physical attributes, including speed, agility, lower limb muscular strength, and explosive power, when compared to the online-offline and online-only participation and non-participation in high-intensity interval training. The analysis further revealed that the online-offline participation exhibited a higher level of effectiveness in explosive power when compared to the online-only participation. Please refer to Table 10 for further details.

**Table 10:** Statistical comparison between groups (LDS method results).

Measure (score)	Group 1	Group 2	Mean 1	Mean 2	Difference	*p*
Self-control	Group A	Group B	109.95	114.833	−4.883	0.118
	Group A	Group C	109.95	102.25	7.7	0.013*
	Group B	Group C	114.833	102.25	12.583	0.000***
BMI	Group A	Group B	95	95.556	−0.556	0.873
	Group A	Group C	95	92	3	0.378
	Group B	Group C	95.556	92	3.556	0.31
Vital capacity	Group A	Group B	83.1	82.944	0.156	0.973
	Group A	Group C	83.1	83.8	−0.7	0.876
	Group B	Group C	82.944	83.8	−0.856	0.853
50-meter sprint	Group A	Group B	71.75	79	−7.25	0.033*
	Group A	Group C	71.75	64.35	7.4	0.026*
	Group B	Group C	79	64.35	14.65	0.000***
Standing long jump	Group A	Group B	72.85	85.333	−12.483	0.041*
	Group A	Group C	72.85	68.6	4.25	0.466
	Group B	Group C	85.333	68.6	16.733	0.007**
Sit-and-reach	Group A	Group B	82.15	86.222	−4.072	0.262
	Group A	Group C	82.15	81.7	0.45	0.898
	Group B	Group C	86.222	81.7	4.522	0.213
800 m/1,000 m	Group A	Group B	70.5	72.667	−2.167	0.463
	Group A	Group C	70.5	72.7	−2.2	0.444
	Group B	Group C	72.667	72.7	−0.033	0.991
Sit-ups/pull-ups	Group A	Group B	38.5	51	−12.5	0.333
	Group A	Group C	38.5	37.9	0.6	0.962
	Group B	Group C	51	37.9	13.1	0.31

*p < 0.05, **p < 0.01, ***p < 0.001.

## 5 Discussion

### 5.1 Online-offline high intensity interval training approach favors improved self-control

By comparing and analyzing the effects of different types of high-intensity interval training on college students’ self-control and physical fitness levels, this study aimed to explore how high-intensity interval training, as an effective form of exercise, can promote the physical and mental health development of college students. The results showed that college students who participated in online-offline high-intensity interval training exhibited significant improvements in self-control, which may be related to the following aspects.

Physiologically, high-intensity interval training can affect the release of neurotransmitters in the brain, such as dopamine and serotonin. Dopamine is associated with the reward system and motivation, and moderate levels of dopamine secretion can increase an individual’s motivation and self-control. Serotonin, on the other hand, helps regulate mood and maintain emotional stability. The release of these neurotransmitters may be facilitated by HIIT exercise, thereby increasing levels of self-control in college students (33).Short-term high-intensity interval training (HIIT) has been demonstrated to exert modulatory effects on motor cortex plasticity and executive function at the neural level. In a study of 32 sedentary women, half of the participants underwent HIIT for a fortnight, and motor cortex plasticity was measured by paired-pulse transcranial magnetic stimulation (ppTMS). This showed changes in short-latency intracortical inhibition (SICI) and intracortical facilitation (ICF) in the HIIT group compared with the control group, as indicated by a decrease in ICF in the HIIT group (34). This finding suggests that short-term HIIT has an effect on motor cortex plasticity. In a separate study that examined the impact of varying doses of HIIT on executive function in university students, both low and medium doses of HIIT were found to enhance the executive function of the participants. The medium-dose group exhibited a more pronounced improvement in this regard (35). The high-intensity nature of HIIT engenders a state of elevated energy expenditure for a brief period, thereby prompting a response from the nervous and endocrine systems. During periods of high-intensity exercise, the prefrontal cortex of the brain is known to be activated. This area is closely related to self-control and executive functions (36). The prefrontal cortex is responsible for higher cognitive functions such as decision-making, planning, and attentional control; self-control is a combination of these functions. When individuals engage in high-intensity exercise, they must constantly suppress instinctive responses such as feelings of fatigue and thoughts of giving up. This process requires the prefrontal cortex to be engaged, thereby exercising self-control. In summary, high-intensity interval training exerts a modulatory effect on motor cortex plasticity and executive function at the neural level, especially on the prefrontal cortex, which has a potential positive effect on executive function, thus enhancing self-control.

The demanding nature of HIIT necessitates that individuals possess both a robust will and determination in order to persevere in the face of adversity and discomfort. This mental challenge has been shown to enhance an individual’s mental toughness and self-efficacy. The successful completion of a high-intensity HIIT session is associated with a marked increase in an individual’s confidence in their own abilities, thereby enhancing their self-efficacy. A study by Eather et al. further demonstrates the efficacy of an 8-week Uni-HIIT program in enhancing physical self-efficacy in a sample of college students, as evidenced by an increase in confidence in their physical abilities among the participants (14). Psychological mechanisms, when doing high intensity interval training, whether active or passive, performing high intensity interval training twice a week has become a specific training goal that they need to achieve. By striving to achieve these goals, their self-confidence and sense of achievement can be increased, which in turn improves self-control. This ability is also transferred to study and life, helping them to better manage their own behavior. At the same time, HIIT can help students relieve stress and regulate their emotions. During high-intensity exercise, the body releases endorphins and other substances that have pain-relieving and pleasurable effects and can improve students’ emotional state. A good emotional state helps to improve self-control and enables them to cope better with various challenges and temptations (37).

Hofmann et al. based on the dual-system model of self-control theory, pointed out that self-control is a result of the interactions between the impulsive system and the control system (38). In summary, according to the dual-system theoretical model, high-intensity interval training (HIIT) has the capacity to enhance self-control through physiological and psychological mechanisms. Physiologically, HIIT can cause neurobiological and structural changes in the brain, and enhance the function of the control system; psychologically, HIIT can enhance willpower, emotional regulation and self-efficacy, and improve the control system’s ability to regulate the automatic system.

### 5.2 Different modes of high-intensity interval training can improve the physical fitness level of university students

The results of the study show that different types of high-intensity interval training have a positive effect on the improvement of college students’ physical fitness and health, especially in terms of physical fitness. In the previous section, we learned that the 50-m sprint tests the development level of students’ speed, agility and nervous system flexibility, the standing long jump mainly measures the development level of explosive force of lower limb muscles and body coordination when jumping forward, and the seated body flexion test reflects the flexibility of joints and muscles. After 8 weeks of high-intensity interval training, Group A’s standing jump and sitting forward flexion scores were significantly improved; Group B’s 50 m and standing long jump scores were significantly improved. This suggests that the students’ developmental levels of speed, agility and nervous system flexibility, the developmental levels of lower limb muscle explosive power and body coordination during forward jumping, and the flexibility of joints and muscles were all improved by the intervention of high-intensity interval training.

A recent study found that HIIT improved racquetball players’ VO2 max, running and repetitive sprint performance, jumping performance and stroke speed during play. HIIT has been shown to significantly improve athletic performance (39). High-intensity interval training (HIIT) has been shown to have a stimulatory effect on the nervous system, thereby increasing the velocity of nerve conduction and muscle contraction. This is achieved by alternating brief periods of high-intensity exercise with intervals of rest, thus enhancing students’ speed performance (40). Significant improvements in physiological variables such as respiratory rate and recovery heart rate in 40 male exercise science students after 8 weeks of high-intensity interval training indirectly suggest that high-intensity interval training may have a positive effect on neurological flexibility, which is closely related to various physiological responses in the body (41).The standing long jump primarily measures the level of development of lower limb muscle explosiveness and body coordination during forward jumps. Researchers studied 100 college-aged students, and while the study focused on the effects of kinesiotape (KT) on standing long jump (SLJ) performance, it also side-stepped the improvement available to college students in terms of lower limb muscle explosiveness. High-intensity interval training can improve lower limb muscle explosiveness by improving muscle strength and endurance, and then improve lower limb muscle explosiveness. At the same time, the training process requires the body to coordinate the movements of different parts, which also helps to improve body coordination. For example, when college students do high-intensity interval training, they need to quickly perform sprinting, jumping and other actions that require the muscles of all parts of the body to work together, thus improving the body’s ability to coordinate (42). It was found that high-intensity interval training methods combined with shoulder and hip flexibility significantly increased VO₂max (38.69 ± 33.19) and lactate threshold (LT) in the control group (*p* < 0.05) but not in the experimental group (*p* > 0.05). This suggests that the combination of high-intensity interval training with site-specific flexibility training has a positive effect on increasing maximal oxygen uptake, although there is uncertainty about the effect on lactate threshold, which indirectly reflects a possible positive effect on overall body function, including flexibility (43).

Different high-intensity interval training methods may have different effects on different physical attributes, but overall, high-intensity interval training is an effective training method for improving the physical health of college students. In practice, personalized high-intensity interval training programs can be developed according to the specific needs and physical conditions of college students in order to maximize their effect on improving physical fitness. The effects of high-intensity interval training are influenced by a variety of factors, including five factors of load intensity, load duration, interval rest intensity, interval rest duration, and number of loads; and five factors of exercise mode, number of sets, duration of multiple sets, intensity between sets, and duration between sets (18). Therefore, the improvement in physical fitness of college students in this paper may be related to these factors of exercise design.

### 5.3 Online-offline high-intensity interval training methods are more advantageous for improving self-control and physical fitness of college students

The online-offline hybrid high-intensity interval training combines the advantages of online and offline instruction, which is more beneficial for self-control and physical fitness improvement of college students.

The online-offline hybrid high-intensity interval training method requires college students to manage and supervise their training effectively. College students need to manage their time reasonably to ensure that they train and study according to the fixed time required by the instructor, which requires them to learn self-control and plan reasonably to avoid procrastination and wasting time. At the same time, through the online platform, students can perform self-assessment based on their own practice data, watch playback videos to understand their own practice movements, evaluate the effect of practice, and make timely adjustments to correct their movements. In addition to the regular Saturday practice time required by teachers, they can play the video in dormitories, libraries and other places with Internet access to memorize the movements and learn and practice repeatedly. This approach can help students develop good self-learning skills (44). Better control of their training progress and effect, thus further improving self-control.

The online platform can provide students with opportunities for social interaction, where they can share their exercise experiences and results with other students, post messages in the forum, and encourage and support each other. This social interaction can increase students’ motivation and enthusiasm for exercise and develop good exercise habits (45). For example, a study of the effects of group high-intensity interval training and continuous aerobic exercise on the quality of life of college students (15) found that subjects in the group fitness class group improved their quality of life significantly more than the individual aerobic exercise group and the control group in the areas of physiology, psychology, social relationships and environment. This suggests that social interactions in group exercise may better motivate students to exercise and improve self-control.

Role model motivation has always played a key role in the study and life of college students. College students are at an important stage of life development and have a strong desire to learn and the ability to imitate. When participating in hybrid online-offline high-intensity interval training, college students may feel difficulties and frustrations. However, when they see their role models successfully overcome these difficulties, they will believe that they are capable of doing the same. This increased confidence will motivate them to participate more actively in training, improve self-control and overcome bad habits such as laziness and procrastination (46). At the same time, the experience of success from role models can motivate students to participate more diligently in training, thereby improving their physical fitness. When students see that role models have achieved significant improvements in physical quality through high-intensity interval training, such as increased muscle strength, speed, agility and flexibility, they will be inspired to actively participate in training. For example, the role models can show their muscle lines, running speed and distance of standing long jump in training, which can be the goals for students to pursue (47).

### 5.4 Training recommendations based on this study

The positive impact of high-intensity interval training on the physical health and self-control of college students can be attributed to the judicious selection of training methods, the formulation of scientifically designed training content, the establishment of effective supervision and feedback mechanisms, and the design of reasonable training programs. These strategies facilitate the enhancement of high-intensity interval training and the improvement of physical health and self-control in college students. The following strategies can be adopted:

#### 5.4.1 Choice of training methods

Diversified high-intensity interval training: The integration of diverse high-intensity interval training modalities has been demonstrated to enhance the overall physical fitness levels of college students. Furthermore, the incorporation of high-intensity interval training into a comprehensive aerobic exercise and muscle strength training program has been shown to enhance the participation and training efficacy of college students.

The present study explores the integration of online and offline high-intensity interval training modalities. The online platform is utilized to provide training instructions, video tutorials and supervision mechanisms, while the actual training sessions and group activities take place offline. The online component encompasses the introduction to the training program, movement demonstration, and the tracking of training progress, among other functions. This enables students to comprehend the training requirements and their progress at any time and in any location. Conversely, the offline segment is designed to facilitate group training activities, thereby enhancing interactivity and teamwork, and fostering heightened student enthusiasm.

#### 5.4.2 Design of training content

Speed, sensitivity and nervous system flexibility training: In order to enhance the developmental level of college students in this domain, the formulation of targeted training programs is recommended. For instance, to enhance speed and explosive strength, training could incorporate squatting actions, punching actions, and explosive training involving jumping actions. Specialized training could include high leg actions, while flexibility and coordination training could involve rope skipping or cross-phase jumping, among other actions. Sensitive training, such as square touchpoint running, in-situ footwork ‘open and close’, etc., can also be employed to exercise the reaction speed of college students and the flexibility of the nervous system.

Lower limb muscle explosive force and body coordination training: Forward and upward jumping exercises are an effective means of training for different distances and heights, such as the standing long jump. Concurrently, they can be combined with deep squatting, jumping squatting and other strength training movements to enhance the explosive force of the lower limb muscles.

Joint and muscle flexibility training: Prior to and following high-intensity interval training, it is imperative to arrange appropriate flexibility training. Such flexibility training can take the form of whole-body stretches, targeting the legs, hips, back, shoulders and other body parts. Examples of such stretches include standing forward bends, side stretches and hip stretches. It is recommended that each movement is held for a predetermined duration and that multiple sets of repetitions are performed. Additionally, during incomplete intervals between movements, stretches such as operatic stretches and marching in place can be incorporated. Furthermore, when engaging in high-intensity interval training, it is recommended to incorporate exercises such as tai chi and aerobic jogging on alternate days. Some of their movements can also be utilized as flexibility training options to assist college students in enhancing their joint range of motion and muscle flexibility.

#### 5.4.3 Reasonable training program

It is vital to establish a suitable frequency and duration for training programs, taking into account the particular circumstances of college students. Generally, 2–3 sessions of high-intensity interval training per week, lasting approximately 30 min each, are considered appropriate. It is important to ensure that training sessions are not excessively prolonged, as this can lead to undue fatigue and the potential for injury. It is also crucial to ensure that the training intensity and the mix of interval time are appropriately balanced to facilitate adequate recovery time after high-intensity training. Gradually increasing the training intensity is another key consideration. During the training process, the intensity should be gradually increased in accordance with the adaptation of college students. The initial phase of training, spanning 5–10 min, is designed to facilitate neurological and muscular adaptation. This phase involves the same movements as formal training, but with intensity that is half or two-thirds of the formal training intensity.

#### 5.4.4 Supervision and feedback of training

The establishment of a monitoring mechanism is imperative to ensure that college students adhere to training and achieve optimal results. Online platforms can be utilized to record training data, facilitating access to real-time feedback for college students. Group activities can also be organized to encourage mutual supervision and support. In addition, coaches or teachers can regularly assess the training of college students and provide guidance and feedback.

The provision of timely feedback and adjustment: During training, feedback should be given to college students in a timely manner so that they can understand their training effect and any issues. Objective feedback can be provided to college students by wearing heart rate bracelets, assessing subjective exercise performance, etc.

## 6 Conclusion

1. High-intensity interval training in different ways has a positive effect on improving the physical fitness and health of college students, and can improve the development of speed, sensitivity and flexibility of the nervous system, the explosive power of the muscles of the lower limbs when jumping forward and the development of body coordination, as well as the flexibility of joints and muscles.
2. Online—offline high-intensity interval training method helps to improve the self-control of college students.
3. The online-offline hybrid high-intensity interval training method is more effective in improving college students’ self-control and the development of speed, agility, and flexibility of the nervous system, as well as the explosive power of the lower limb muscles when jumping forward and the development of body coordination ability, compared to online-only participation in high-intensity interval training and non-participation in high-intensity interval training.

## 7 Limitations

High-intensity interval training is of great importance in the study of self-control and physical fitness levels in college students, and although the current study was effective, it has some limitations, while the direction of future research is worth exploring in depth.

The smaller sample size may mean that the results of the study are not representative enough to be generalized to a larger group of college students, thus affecting the generality of the findings. In the future, consideration will be given to sampling students from different regions, subjects and years to cover a wider range of student group characteristics. This will provide a better understanding of the differences in the effects of different types of high-intensity interval training in different groups of students, and provide a basis for the development of more targeted training programs.

Improvements in physical fitness often require a long-term commitment to training, while improvements in self-control can also be a gradual process. In a relatively short period of time, only some short-term changes may be observed and it is difficult to accurately assess the long-term effects. Future studies will consider long-term follow-up studies to monitor the long-term effects of high-intensity interval training on self-control and physical fitness in college students. For example, multiple time points will be used for data collection to gain a more comprehensive understanding of trends in training effects.

Social desirability effects: It may be beneficial for future studies to consider incorporating experimental methods (e.g., initiation paradigm) or implicit measures (e.g., IAT Implicit Association Test) to provide more objective assessments of the variables of interest.

In addition, more factors need to be considered: college students have large individual differences in their physical condition, athletic background and psychological characteristics. Future studies can take these individual differences into account, such as group studies based on students’ physical fitness, athletic ability, self-control level, etc., to understand the differences in the effects of high-intensity interval training on different types of students.

Psychological factors: Self-control is not only influenced by physiological factors, but is also closely related to psychological factors. Future studies can further explore the role of psychological factors in high-intensity interval training, such as motivation, willpower, emotional state, etc., which can provide psychological intervention strategies to improve training effectiveness.

## Data Availability

The original contributions presented in the study are included in the article/supplementary material, further inquiries can be directed to the corresponding author.
